# ECONOMIC INEQUALITY AND FUTURE THOUGHT AMONG OLDER ADULTS

**DOI:** 10.1093/geroni/igz038.2750

**Published:** 2019-11-08

**Authors:** Erin M Adamson, David J Ekerdt, Erin M Adamson

**Affiliations:** University of Kansas, Lawrence, Kansas, United States

## Abstract

Cumulative advantage theory sees inequality extending into later life. Does inequality also extend to the imagination of one’s future and desires for life yet to come? We draw from semi-structured interviews with 55 individuals residing in Midwest cities to explore differences in talk about finances and expectations for longevity, fulfillment, and “active” pursuits. Addressing personal futures, participants’ desires for longevity varied. Lower SES individuals discussed not having expected to live to their current age (or much beyond it) and not wanting to live a long time. Higher SES individuals, by contrast, expressed confidence that they could afford care no matter how long they lived. Higher SES participants often described future leisure goals whereas lower SES participants tended not to name leisure goals, or they named activities they desired but could not afford. For low SES individuals, active pursuits also were limited by diseases disproportionately affecting poor Americans, such as diabetes.

